# Electrospun Polycaprolactone–Gelatin Fibrils Enabled 3D Hydrogel Microcapsules for Biomedical Applications

**DOI:** 10.3390/jfb16030085

**Published:** 2025-03-02

**Authors:** Felix Tettey-Engmann, Thakur Sapkota, Sita Shrestha, Narayan Bhattarai, Salil Desai

**Affiliations:** 1 Department of Chemical, Biological and Bioengineering, North Carolina A&T State University, Greensboro, NC 27411, USA; fatettey@aggies.ncat.edu (F.T.-E.); tsapkota@aggies.ncat.edu (T.S.); sshrestha2@ncat.edu (S.S.); nbhattar@ncat.edu (N.B.); 2 Department of Industrial and Systems Engineering, North Carolina A&T State University, Greensboro, NC 27411, USA; 3 Department of Applied Science and Technology, North Carolina A&T State University, Greensboro, NC 27411, USA

**Keywords:** 3D alginate hydrogel, gelatin, microcapsules, nanofibrils, polycaprolactone, electrospraying

## Abstract

Microcapsules provide a microenvironment by improving the protection and delivery of cells and drugs to specific tissue areas, promoting cell integration and tissue regeneration. Effective microcapsules must not only be permeable for micronutrient diffusion but mechanically stable. Alginate hydrogel is one of the commonly used biomaterials for fabricating microcapsules due to its gel-forming ability and low toxicity. However, its mechanical instability, inertness, and excessive porosity have impeded its use. Embedding nanofibrils in the alginate hydrogel microcapsules improves their biological and mechanical properties. In this research, electrospun composite nanofibers of PCL–gelatin (PG) were first fabricated, characterized, and cryoground. The filtered and cryoground powder solution was mixed with the alginate solution and through electrospray, fabricated into microcapsules. Parameters such as flow rate, voltage, and hydrogel composition, which are critical in the electrostatic encapsulation process, were optimized. The microcapsules were further immersed in different solvent environments (DI water, complete media, and PBS), which were observed and compared for their morphology, size distribution, and mechanical stability properties. The average diameters of the PG nanofibers ranged between 0.2 and 2 μm, with an average porosity between 58 and 73%. The average size of the microcapsules varied between 300 and 900 μm, depending on the solvent environment. Overall, results showed an improved alginate 3D hydrogel network suitable for biomedical applications.

## 1. Introduction

Microencapsulation technology has emerged as a cornerstone in various fields for creating robust 3D platforms for cell culture study [1]. Before 3D platforms, electrospinning technology was vital for its promotion of biocompatibility; however, its 2D format limits overall cell proliferation [2,3]. Direct cell injection to target areas involves a risk of exposing cells to high shear stresses, resulting in cell leakage and triggering an unintended host–immune response if biomaterial shielding is not provided. Encapsulation methods improve the protection and delivery of cells and drugs to targeted tissue locations, facilitating cell integration and, consequently, tissue regeneration and repair. Hydrogels are used widely in encapsulation for the production of microcapsules due to their many desirable properties [4]. For embedded cells, hydrogel offers a highly hydrated microenvironment that is capable of presenting cellular, biochemical, and physical stimuli that direct cellular activities like migration, proliferation, and differentiation [5]. Also, the hydrogel’s soft and compliant qualities lessen the mechanical or frictional discomfort to the surrounding tissue. Furthermore, its hydrophilic qualities nearly eliminate interfacial tension with surrounding tissues and fluids, which reduces protein adsorption and cell adhesion. Hydrogels offer low-molecular-mass nutrients and metabolites and a high degree of permeability. Among the plethora of hydrogels available for microencapsulation, alginate, a naturally occurring polysaccharide extracted from brown algae, possesses unique encapsulation properties that make it suitable for various biomedical applications [6]. Alginate is easily processable into three-dimensional (3D) scaffolding materials like microcapsules, which are beneficial for diverse applications [7]. Alginate can form hydrogels through ionic crosslinking with divalent cations such as calcium, resulting in the formation of spherical microcapsules [8]. These microcapsules can be fabricated using techniques such as extrusion, emulsification, or electrostatic droplet generation, offering control over size, shape, and mechanical properties [9]. Despite the significant advancements, challenges remain in optimizing the effectiveness of alginate microcapsules for biomedical applications. The mechanical instability of pure alginate hydrogels may not be sufficient for certain applications [5]. Also, alginate’s limited protein adsorption capacity, leading to poor cell adherence on the surface and uncontrollable degradation, is a limitation and has to be improved [10]. The incorporation of reinforcing polymeric electrospun fibrils with alginate microcapsules offers several advantages in biomedical applications [11]. Firstly, the fibrous structure of the nanofibrils mimics the native extracellular matrix (ECM), promoting cell attachment, proliferation, and differentiation and improving the mechanical stability of alginate. Secondly, the porous nature of the scaffold facilitates nutrient diffusion and waste removal, critical for cell viability and overall tissue regeneration. Thirdly, the encapsulation of bioactive factors within alginate microcapsules enables controlled release, allowing the spatiotemporal modulation of cellular behavior and tissue regeneration. Furthermore, the biocompatibility and biodegradability of both polymeric fibrils and alginate minimize opposing immune reactions and facilitate scaffold remodeling over time [11]. Polymeric nanofibers, before being fibrils, are foremost produced utilizing electrospinning. Electrospinning is a technique that employs an electric field to a polymer solution or melt, resulting in fibers drawn from a droplet or a jet of polymer solution and collected on a grounded substrate, resulting in a non-woven mat or scaffold [12]. Electrospinning is used to fabricate nano- to microscale fibers from a variety of materials, and it enables precise control over fiber diameter, orientation, and porosity, making it well suited for various applications. Before encapsulation, the electrospun scaffolds are further broken into particles using cryogrinding technology [13]. This technique involves cooling or subjecting the material up to or lower than glass transition temperature with liquid nitrogen, followed by mechanical grinding, producing fine powders with unique properties, opening up new possibilities [14]. This technique can effectively grind materials that are difficult to process at ambient temperatures, thus eliminating much of the quality hassles of traditional grinding [15]. In the context of fiber processing, cryogrinding offers several advantages. The extremely low temperatures used in cryogrinding (typically using liquid nitrogen at −195.6 °C) cause materials to become more brittle [15], making them easier to grind into fine particles compared to room temperature grinding. The low temperatures used in cryogrinding prevents the thermal degradation of heat-sensitive materials, which is particularly important for pharmaceuticals and certain polymers [16]. Recent studies have demonstrated the effectiveness of cryogrinding in processing various fibers. For example, research on cotton fiber cellulose showed that cryogrinding resulted in decreased particle size and altered physicochemical properties [17]. Similarly, studies on natural fibers such as sisal and orange tree fibers have explored the effects of cryogrinding on morphological changes, crystallinity, and stability [18]. Several polymers, including synthetic and natural materials, have been electrospun to produce scaffolds for biomedical applications [19]. Polycaprolactone (PCL), a biodegradable and biocompatible synthetic polymer, offers mechanical stability and structural integrity. As a result, alginate combined with PCL has been used in applications that demand mechanical strength [20,21]. PCL and alginate, when used alone, increase hydrophobicity, making them challenging for cell attachment and bioactivity [22]. To improve on the hydrophilicity of PCL, natural polymers such as chitosan and gelatin are introduced to alter the chemistry of PCL to improve its hydrophilicity [23]. The combination of alginate with other materials such as chitosan, PEG, biosilica, ceramics, bio glasses, bone morphogenetic protein-2, and proteins like collagen or gelatin has shown enhanced properties like biocompatibility, porosity, mechanical strength, and osteogenic differentiation [24,25]. Gelatin, a natural biopolymer derived from collagen, imparts bioactivity and cell interactivity due to its natural protein composition fostering a conducive environment for cellular proliferation and tissue regeneration [26,27]. By harnessing the synergistic interplay between natural and synthetic materials, researchers have endeavored to engineer hydrogel scaffolds with tunable properties, aiming to mimic the extracellular matrix (ECM) and thus advance applications ranging from tissue engineering to drug delivery [24,28]. These polymeric properties, when synergistically introduced into the hydrogel during encapsulation, can help overcome the mechanical, physicochemical, and biological limitations of soft hydrogels like alginate.

To prove this, we performed this study by first fabricating electrospun composite nanofibers of PCL–gelatin (PG) at different weight ratios named PG-00, PG-10, PG-20, and PG-50. The respective numbers represent the percentage of gelatin with PCL. The electrospun PG samples were characterized by their physicochemical, mechanical, and biological properties. After successful characterization, the electrospun nanofibers were cryoground using a liquid-nitrogen-containing cryogenic impact grinder. The filtered cryoground solution was mixed with the alginate solution. Through electrospray, the microcapsules were fabricated from the mixture. The hydrogel composition, flow rate, and voltage parameters were explored and optimized. The alginate–PCL gelatin (APG) microcapsules were immersed in different solution environments, DI water, complete media, and PBS after fabrication and characterized for specific days. In summary, the utilization of PCL and gelatin in 3D alginate hydrogel microcapsules represents a significant advancement, offering superior mechanical properties, enhanced cellular interactions, versatility in encapsulating bioactive agents, and facilitated tissue integration. These attributes position PCL–gelatin with alginate microcapsules as promising candidates for a myriad of biomedical applications, ranging from controlled drug delivery to organoids and regenerative medicine, paving the way for innovative therapeutic interventions and improved patient outcomes.

## 2. Materials and Methods

### 2.1. Preparation of Polycaprolactone-Gelatin Electrospun Fibers

Solutions for electrospinning were prepared as described in the literature [29,30] for the fabrication of the two-dimensional (2D) electrospun PCL–gelatin (PG) nanofiber mats. Briefly, PCL pellets (Mw 80,000, Sigma-Aldrich, St. Louis, MO, USA) and gelatin powder (Product G9391-100G, Sigma-Aldrich, St. Louis, MO, USA) were each dissolved in 2,2,2 trifluoroethanol (TFE) (Alfa Aesar, Ward Hill, MA, USA) at 10% (*w*/*w*) with constant magnetic stirring for 12 h at room temperature. In total, 5 mL of blend solutions of PCL and gelatin at different ratios were made, and before electrospinning, each solution was subjected to vortex mixing for 5 min. TFE had a pH ranging from 5.0 to 7.5 (10% *v*/*v* soln.) and a boiling point of 77.0 °C to 80.0 °C. With regard to temperature, consistent room temperature (20–25 °C) was considered to prevent phase separation before and during electrospinning.

The various proportions of PG solution prepared for electrospinning are shown (Table 1). Authors chose these compositions based on previous experiments that suggested significant changes in the properties of these compositions using a synthetic and natural polymer composite blend [31,32]. Also, trial experiments during electrospinning at different compositions were executed for this work, and based on the outcomes, we determined what ratio samples would be used going forward.

A customized built-in horizontal electrospinning setup was exploited. In total, 5 mL of the vortexed PG solution was collected into a 10 mL BD-Luer-Lok syringe capped with an 18-gauge diameter hypodermic tip and an aluminum-covered rotating collector placed at 12 cm from the tip of the needle. The syringe with the PG solution was held firmly to a syringe pump (Model 78-01001, Fisher Scientific, Pittsburgh, PA, USA), which operated with a +15 kV high-voltage supplier (Model CZE100PN30, Spellman High Voltage Electronics Corporation, Hauppauge, NY, USA) attached to the needle at a constant flow rate of 1.0 mL/h. The use of the high voltage mandates earth grounding. Each testing solution was pumped independently, and the fibers collected on the aluminum foil were peeled for further analysis.

#### 2.1.1. Surface Morphology Analysis of PCL–Gelatin Electrospun Nanofibers

Dry nanofiber samples were analyzed using a scanning electron microscope (SEM) (JEOL JSM-IT800HL Inc., Peabody, MA, USA). In summary, the electrospun PG fibrous samples were cut into 2 mm^2^ strips, attached to an aluminum stub using a double-sided conductive tape and sputter-coated with gold–palladium using a coating system (Leica EM ACE200, IL, USA) for 30 s at a coating depth of 5 nm at 15 mA. Samples were visualized at a 3 kV accelerating voltage. The fiber’s size distribution was analyzed through SEM images with the use of ImageJ 1.53c software (NIH, Bethesda, MD, USA). The diameter length was converted to pixels with the help of a scale bar. Fifty individual fibers of PG samples (n = 3) were measured in pixels. The average size and standard deviation were calculated based on converted ImageJ data. Furthermore, the electrospun mats were cut into rectangular shapes, and their respective width, thickness, and length were measured to calculate the volume (V). The weight of the electrospun dry samples (m) was carefully measured using an analytic balance by the accuracy of 0.001 g, which was further used to estimate the surface area and surface area per unit weight of the samples. The density of every sample was calculated from its weight divided by volume. A digital micrometer screw gauge was used to determine the scaffolds’ thickness. The electrospun PG samples’ porosity (ϕ) was determined by comparing the apparent or mean density (ρ^a^) to the bulk density (ρ^b^) of the scaffolds using the equation as adopted in a previous publication [33].

#### 2.1.2. Chemical Composition Analysis

The Fourier transmission infrared (FT-IR) spectra of the samples were measured utilizing an FT-IR spectrometer (Agilent 670, Santa Clara, CA, USA). The FTIR spectra of PG were recorded by the accumulation of 32 scans in the range of 3700–600 cm^−1^, with a spectral resolution of 4 cm^−1^.

#### 2.1.3. Surface Hydrophilicity and Surface Energy Determination

The wetting properties of the PG samples were evaluated by measuring the static contact angle between the water drop and the PG sample surface using a Drop Shape Analyzer DSA 25E (Kruss, Hamburg, Germany) at room temperature. The setup for this test included a vertical clamp containing the droplet syringe, a sample stage, one end with a focused light source, and the other with a camera end connected to the computer system that captured the images. An approximately 2 μL volume water drop at a rate of 2.67 μL s^−1^ was placed on the sample surface with a micro syringe. The contact angle measurements were captured at 10 s and 30 s, and averages were taken to represent the water contact angle of the sample. Samples with higher contact angles are said to possess low-energy surfaces whereas those with lower contact angles are said to possess high-energy surfaces.

#### 2.1.4. The Mechanical Property Analysis

The mechanical performance of electrospun samples was determined using a uniaxial tensile test with a 500 N load cell under a crosshead rate of 10 mm/min. The thickness of the nanofibrous mat was measured using a digital micrometer, and the samples were cut into 40 × 15 mm^2^ rectangles and situated in the grips by a gauge length of 20 mm. Each electrospun composite mat (n = 5) was stretched until break point. Finally, a stress–strain plot, ultimate tensile stress (UTS), and elongation at break (EB) were measured from the load-extension data.

#### 2.1.5. Cell Culture Study on PG Fibrous Scaffolds

##### Cell Seeding to PG Scaffolds

NIH-3T3 mouse fibroblast cell lines (American Type Culture Collection, (ATCC) Cell Line Bank 1658, Manassas, VA, USA) were cultured with complete media (Dulbecco’s modified Eagle’s medium (DMEM)), supplemented with 10% fetal bovine serum (FBS), 1% penicillin and streptomycin at 37 °C under 5% CO_2_ and 95% humidified. Upon 80% confluency, cells were dissociated by trypsinization (trypsin-EDTA, Gibco, thermos Fisher Scientific) before seeding on electrospun scaffolds. The different electrospun scaffolds (n = 3 per sample) with dimensions of 1.2 × 1.2 cm^2^ were fitted on the circular glass coverslips and fixed into the 48-well plates (Thermo-fisher scientific Waltham, MA, USA) and sterilized under UV for about 3 h. The scaffolds were then washed with 70% ethanol followed by washing with phosphate-buffered saline (PBS). The cells at a density of 3 × 10^4^ were seeded on each scaffold by pipetting onto the center of the scaffold and cultured in the incubator for 1 to 3 days. The cells were nourished with fresh media on the second day. However, for cell viability and toxicity studies, media extracts were collected each day and stored at 4 °C. These studies are described further in Section Cell Viability and Toxicity Analysis.

##### Cell Viability and Toxicity Analysis

NIH-3T3 cell viability was examined with Alamar Blue (AB) colorimetric assay (Thermo-fisher scientific, Waltham, MA, USA), as described in previous works [34,35]. Briefly, the cell-laden fibrous scaffolds were treated with 10% AB reagent in cultured media and incubated for 4 h. Assay solutions were transferred to 96-well plates to measure fluorescence (530 nm excitation and 590 nm emission). The cell toxicity of the PG scaffolds was evaluated using the Pierce Lactose Dehydrogenase (LDH) assay kit (Thermo-fisher scientific, Waltham, MA, USA) with the manufacturer’s instructions and also with reference to previous works [29,36]. In summary, 50 µL of stored sample media (n = 3) was transferred to a 96-well plate and mixed with a 50 µL reaction mixture. The well plate was then covered with aluminum foil to prevent the reactions’ exposure to light and incubated at room temperature for 30 min. Then, 50 µL of a stop solution was added to each well to stop the reaction, and the absorbance of the samples was measured at 490 nm and 680 nm using a microplate reader (CLARIOstar Plus, BMG LABTECH Inc., Cary, NC, USA).

The adhesion and survivability of the cells on the scaffolds were examined with a live/dead assay kit (Perkin Elmer LLC via AOPI Staining Solution; Thermo-fisher scientific, Waltham, MA, USA), in accordance with the company’s protocol. The live and dead cells stained in green and red color, respectively, were visualized and captured with an Olympus I × 83 microscope incorporated with Olympus cell Sens Dimension software version 3.2 (Olympus Corporation, Shinjuku, Tokyo, Japan). Live and dead cells were counted from the fluorescence images using ImageJ 1.53c software.

#### 2.1.6. Preparation of Polycaprolactone–Gelatin Fibrils

The electrospun nanofiber mesh of PG was subjected to a cryogrinding process to obtain a ground powder of PG. The cryo-equipment, a cryogenic impact grinder (Model 6775 Freezer/Mill, Fisher Scientific, Pittsburgh, PA, USA), contains a small polycarbonate grinding vial that grinds up to 5 g of a sample. The sample is pulverized by the magnetically shuttled steel impactor that goes back and forth against the steel end plugs. Liquid nitrogen is utilized in the operation to keep the sample cold, preserving its integrity [37]. Before starting the run, the PG nanofiber mats were cut into small squares of approximately 1 × 1 mm^2^, weighed, and kept in isopropanol for 20 min. This was to soften the nanofibers for easy grinding. These wet fibers were squeezed to drain out excess alcohol and placed in the polycarbonate grinding vial with a magnetically shuttled steel impactor and a metal bar at both ends. The polycarbonate tube was then placed inside the cryogrinding apparatus, which was then filled with liquid nitrogen (~4–5 L) and closed. The cryogrinding machine was set automatically to grind 6 times at a rate of 12 cycles per second (CPS), with each cycle running a 5-min precool, a run time of 1 min, and a cool time of 1 min. The applied impact bar frequency was 14 Hz. After finishing and returning to room temperature, water was used as a medium to filter ground PG solutions through a 70 μm mesh sieve. This filtered solution was observed for its dispersion effectiveness. The filtered cryoground solution was stored in a freezer and later lyophilized to achieve a relatively small fine-dried powder content of the PG for further studies. Lyophilization ensured the removal of all the fluid, leaving the powder. Before microencapsulation, the powder was mixed with water and vortexed for effective distribution. This is identified as the PG solution.

### 2.2. Three-Dimensional Hydrogel Microcapsule Fabrication

Notably, 2% (*w*/*v*) alginate solution was prepared by dissolving 2 g of alginate powder in 100 mL of 1X Hanks’ Balanced Salt Solution (HBSS) buffer, which was subjected to magnetic stirring for 12 h for homogeneity. A CaCl_2_ solution for crosslinking with the alginate solution was prepared by dissolving 16.665 g of CaCl_2_ powder in 1000 mL of DI water. The PG solution, before mixing with the alginate solution, was placed in an ultrasonic bath (PS-10A Jeken ultrasonic cleaner bath, Jeken, Dongguan, China) for 10 min. For a more effective homogeneity, the PG solution was subjected to probe sonication for an additional 2 h. After sonication, the PG solution at different weight ratios was mixed with the alginate solution and drawn into a syringe fitted with a 24-gauge needle for electrospraying, using predefined setup conditions, as utilized in previous works [38,39]. Microcapsules were fabricated by utilizing the co-axial electrohydrodynamic atomization method. The as-prepared alginate–PG nanofibrils colloidal was loaded in a syringe set on a vertically mounted thermofisher syringe pump for the droplets ejected to fall onto the 150 mM CaCl_2_ solution. The alginate–PG (APG) microcapsules were fabricated employing a 5 kV voltage supply and an 18 mL/h pump flow rate. The distance from the tip to the surface of the CaCl_2_ was fixed at 25 mm, and the ground electrode was immersed in the CaCl_2,_ receiving a bath before initiating the run. After complete exhaustion of the solution from the syringe, the setup was turned off, and the APG microcapsules that were produced were allowed to settle and washed twice with DI water. The microcapsules were made maintaining a fixed alginate/fiber ratio of 70/30 while varying the PCL–gelatin composition within the fibers. The as-produced microcapsules were distributed in DI water, complete media, and PBS solutions for days 1 to 7 and later characterized for morphology, size, and stability. Details of the microcapsule compositions are shown (Table 2). The alginate-only microcapsules without both PCL and gelatin (PG) nanofibrils denoted as AAA were the controls.

#### 2.2.1. Characterization of APG Microcapsules

##### Morphology and Size Distribution of the Microcapsules

The morphology of the fabricated APG microcapsules in different solution environments was observed using an optical microscope (EVOS^®^ FL Core Digital Inverted, life technologies, Bothell, WA, USA). Microcapsules (n = 50) were sampled for the diameter using Image J 1.53c software.

##### Mechanical Properties’ Analysis of the APG Microcapsules

The testing instrument (Micro Tester—Cell Scale Biomaterials Testing, Waterloo, ON, Canada) was employed for the stability test of the microcapsules according to a prior publication [40]. Briefly, the instrument is equipped with a water bath for the hydrated samples. A two parallel plate compressive system was used to measure the mechanical properties of the microcapsule by applying a compressive force between the two parallel plates of each sample. A circular tungsten microbeam with a length and diameter of 58 and 0.5588 mm, respectively, was used to apply a constant force to microcapsules (n = 5 per composition), which were kept at 37 °C in DI water. Using an incorporated image analysis module in a microscale test system, the force (μN) and displacement (μm) were measured. Using the company’s software, microcapsules were crushed to a maximum of 30% of their initial diameter for 20 s, and force–displacement curves were recorded on the dashboard. The raw data generated from the experiment were further explored for Young’s modulus (YM) and other properties.

### 2.3. Statistical Analysis

All data were analyzed using one-way analysis of variance (ANOVA) for significance with OriginPro software 2023 Version (Origin Lab, Northampton, MA, USA). Post hoc Tukey’s test was utilized with ANOVA for multiple comparisons and presented as mean ± standard deviation (S.D). The α-value was set to 0.05 and 0.01, and *p*-values less than 0.05 and 0.01 were considered statistically significant.

## 3. Results

### 3.1. Surface Morphology Analysis of Electrospun PG Nanofibers

**Figure 1:**
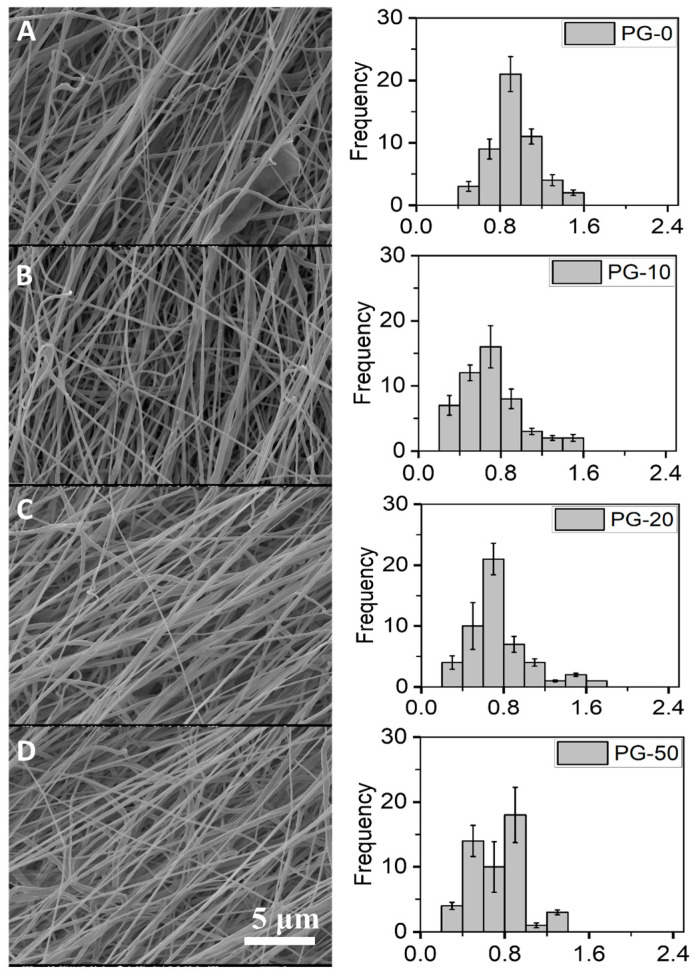
Surface morphology and diameter distributions of PG scaffolds. (**A**–**D**) represent the SEM images of PG-00 to PG-50, respectively, and their corresponding histogram showing the fiber diameter distribution of the as-prepared PG-00 to PG-50 samples. Scale bar: 5 μm with 1.50 k magnification.

The fibrous morphology and diameter distributions of the electrospun PG nanofibers are represented (Figure 1). Smooth and uniform fibers were recorded for all PG nanofibers. The diameters of the PG nanofibers ranged between 0.2 and 2 μm with the average diameter of PG-00, PG-10, PG-20, and PG-50 estimated as 1.2 ± 0.35, 0.81 ± 0.3, 0.77 ± 0.11, and 0.73 ± 0.37, respectively.

The thicknesses of the PG-00, PG-10, PG-20, and PG-50 scaffolds were estimated in millimeters (mm) as 0.155 ± 0.0063, 0.149 ± 0.0048, 0.163 ± 0.0041, and 0.194 ± 0.0093, respectively. Respective sample thickness, surface area, surface area per unit weight, and porosity were determined and are summarized (Table 3). From the table, there was a distinct increase in fiber thickness as incremental gelatin was added to PCL, and as such, PG-50 recorded the highest fiber thickness at 0.194 ± 0.0093. PG-10 did not show an increase in fiber thickness. This could be explained as a result of the non-homogeneity of the gelatin distribution in the fiber.

### 3.2. FTIR Spectra Analysis of PG Nanofibers

**Figure 2:**
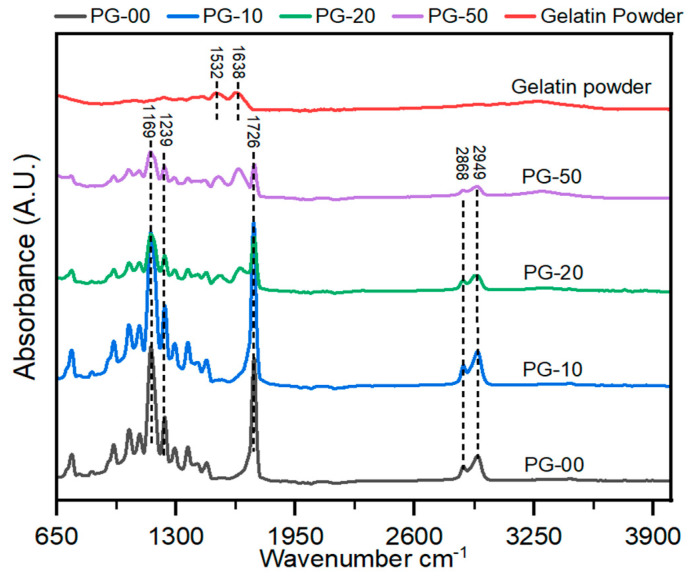
Analysis of the chemical composition of the PG electrospun nanofiber. FT-IR spectra of gelatin powder and PG scaffolds. All peaks at 1726 show PCL, and those between 1532 and 1638 indicate gelatin.

The FTIR spectra for the different testing material samples are shown (Figure 2). The PCL-only scaffold (black spectra) revealed characteristic peaks of PCL at peak 1726 cm^−1^ for the stretch of CO in the ester groups and 1239 cm^− 1^ and 1169 cm^− 1^ for the asymmetric and symmetric stretching vibrations of C–O–C. These representative peaks of PCL are similar to earlier reported work [41]. The gelatin powder (red spectra) revealed characteristic peaks of gelatin at peaks 1532 and 1638. The gelatin powder showed characteristic peaks at 1532 and 1638, which are associated with gelatin. Similalr peaks in PG samples, confirms the presence of gelatin in the PG scaffolds.

### 3.3. Surface Wettability Analysis of PG Samples

From the contact angle measurement of the fibrous scaffolds, the average contact angles with their standard deviation of PG-00 to PG-50 were recorded as 122 ± 6.14, 78 ± 3.42, 72 ± 5.11, and 61 ± 3.88, respectively. This indicates that as gelatin content increases, the contact angle decreases; as such, PG-50 recorded the lowest contact angle measure, whereas PG-00 recorded the highest contact angle reading. The contact angle measurement summary which determines the surface wettability is summarized (Table 4).

### 3.4. Mechanical Properties of PG Scaffolds

**Figure 3:**
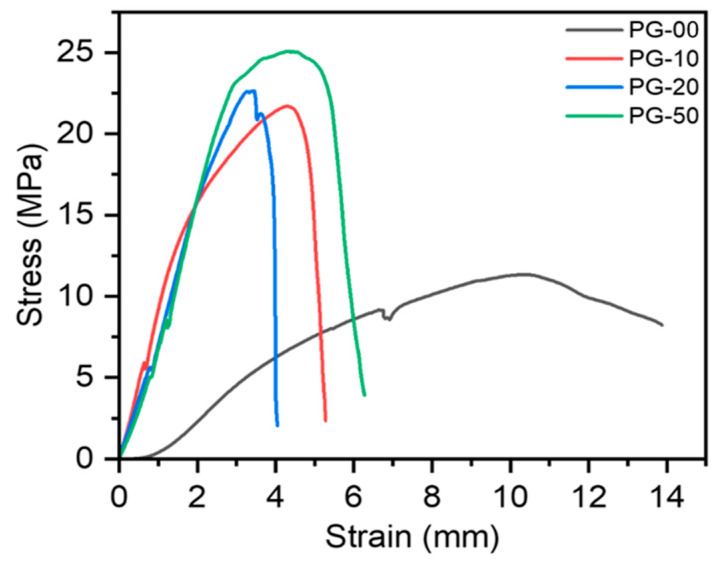
Mechanical properties of PG scaffolds investigated by tensile testing. Stress–strain curves of PG samples generated from the force–displacement runs.

The exposure of these materials to the physiological environment requires their stability to withstand external forces; as such, the mechanical properties were determined. The mechanical properties of the PG scaffolds expressed in stress–strain curves are shown (Figure 3). These curves were generated from a further analysis of the raw data gathered from the force–displacement curves generated after the test. The stress–strain curves show that the gelatin-containing scaffolds have better stiffness than the pure PCL (PG-00). The summary of the quantitative data from the mechanical testing is also shown (Table 5).

### 3.5. In Vitro Cytocompatibility and Cell Proliferation Analysis

**Figure 4:**
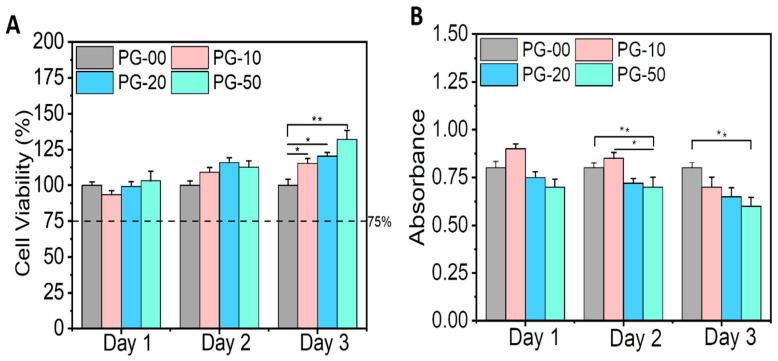
In vitro cytotoxicity testing. (**A**) represents the cell viability plot of the PG samples with the NIH-3T3 cells utilizing the Alamar Blue assay, and (**B**) represents the toxicity plot of the PG samples with the NIH-3T3 cells utilizing the LDH assay. Statistical analysis was performed using the one-way ANOVA with Tukey’s post hoc method, and the data are expressed as the mean ± SD; n = 3 per group for cell viability test (where * *p* < 0.05 and ** *p* < 0.01).

**Figure 5:**
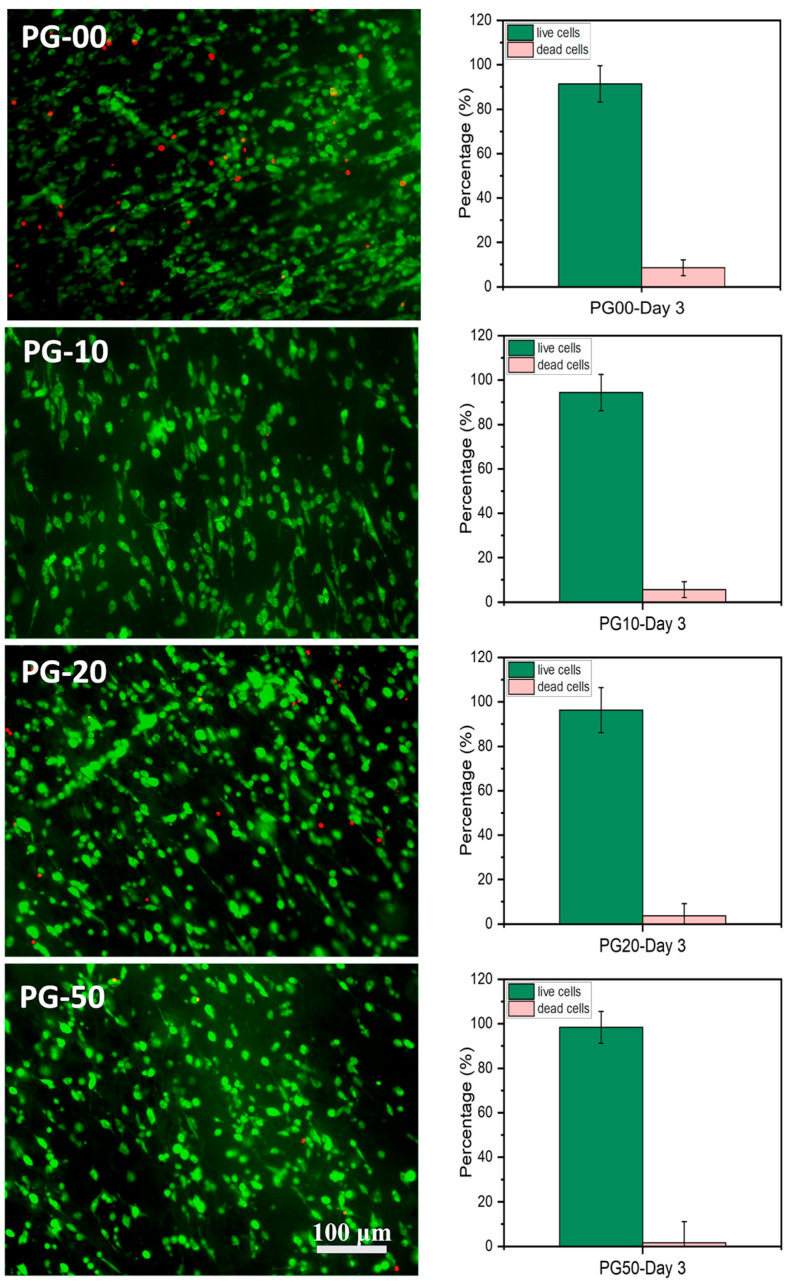
Cell microscopy measure. (**Left**) Fluorescence microscopy images of live (stained green) and dead (stained red) NIH-3T3 fibroblasts cultured on the PG scaffolds on day 3 using acridine orange/propidium iodide (AOPI) dye (scale bar = 100 μm). (**Right**) Histograms show the counted percentage of live and dead cells in the corresponding fluorescence images using the ImageJ software (*n* = 5) of PG-00 to PG-50, respectively.

The biocompatibility study of the PG sample was undertaken utilizing Alamar Blue assay to determine cell viability and LDH assay to quantify cell toxicity. Figure 4A clearly shows the viability of all materials with cells regardless of the day in consideration. There was no significant difference in cell proliferation on day 1 and day 2 in all samples, but on day 3, the NIH-3T3 cells proliferated significantly on PG-10, PG-20, and PG-50, when compared with PG-00. Figure 4B shows the corresponding toxicity plot of the samples with cells. The survivability and proliferative effect of the cells on the fabricated scaffolds were established by the live/dead staining kit. The live/dead fluorescence images are shown (Figure 5).

### 3.6. Cryogrinding of Electrospun Nanofibers

**Figure 6:**
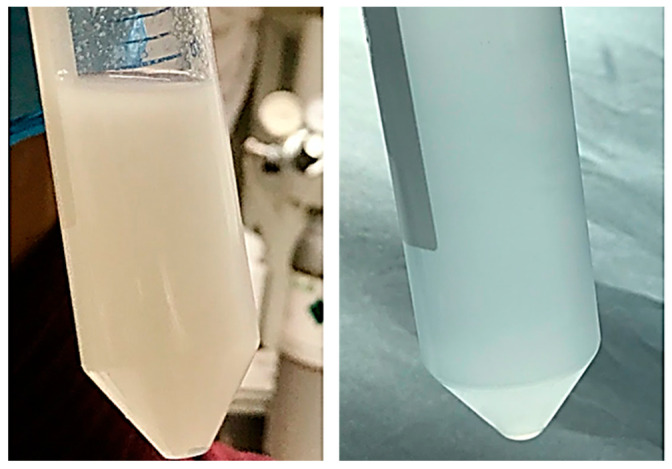
Dispersion of cryoground PG. Left is the slightly opaque dispersion of cryoground PG in water before filtration, and right is the slightly transparent dispersion after filtration through a 70 µm filter.

**Figure 7:**
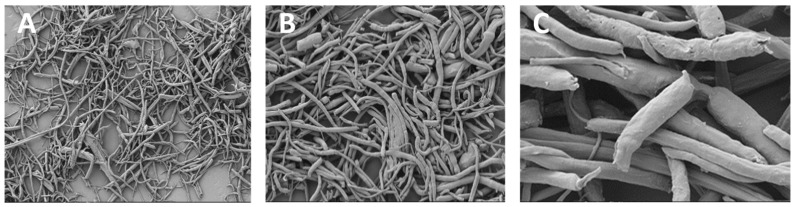
SEM images of cryoground PG nanofibers under (**A**) 500 X magnification and a scale bar of 20 µm; (**B**) 1.00 KX magnification and a scale bar of 10 µm; (**C**) 5.00 KX magnification and a scale bar of 1 µm.

**Figure 8:**
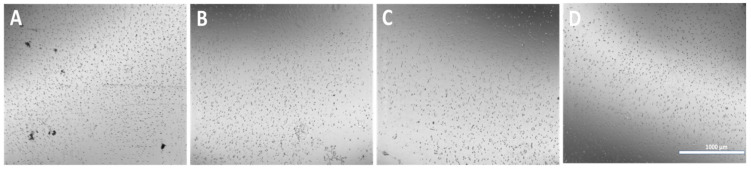
Optical images of cryoground nanofibers dispersed in DI water. (**A**–**D**) represent PG-00, 10, 20, and 50, respectively, after 2 h of probe sonication for the dispersion of particles. (Scale bar = 1000 μm).

The dispersion of the cryoground PG in water before and after filtration via a 70 µm filter is shown (Figure 6). After filtration, it showed a more transparent view as compared to before filtration. A drop of the after-filtrate was suspended on aluminum foil and allowed to dry. Figure 7 further shows SEM images at different magnifications of the dry cryoground PG nanofibers’ morphology. It was earlier observed that prior to sonication, a drop of filtrate from all samples contained moving chunks of fiber when viewed with the optical microscope. It was further observed that highly hydrophobic PCL, which is represented as PG-00, had more chunks, which hindered a better fiber dispersion significantly more than other fiber samples. A 2 h ultrasonication process was followed to achieve a better dispersion of the powder in DI water, with results shown (Figure 8). Overall, the adoption of the ultrasonication process resulted in an ultra-pure fiber solution before alginate mixing.

### 3.7. Microcapsules’ Fabrication and Characterization

**Figure 9:**
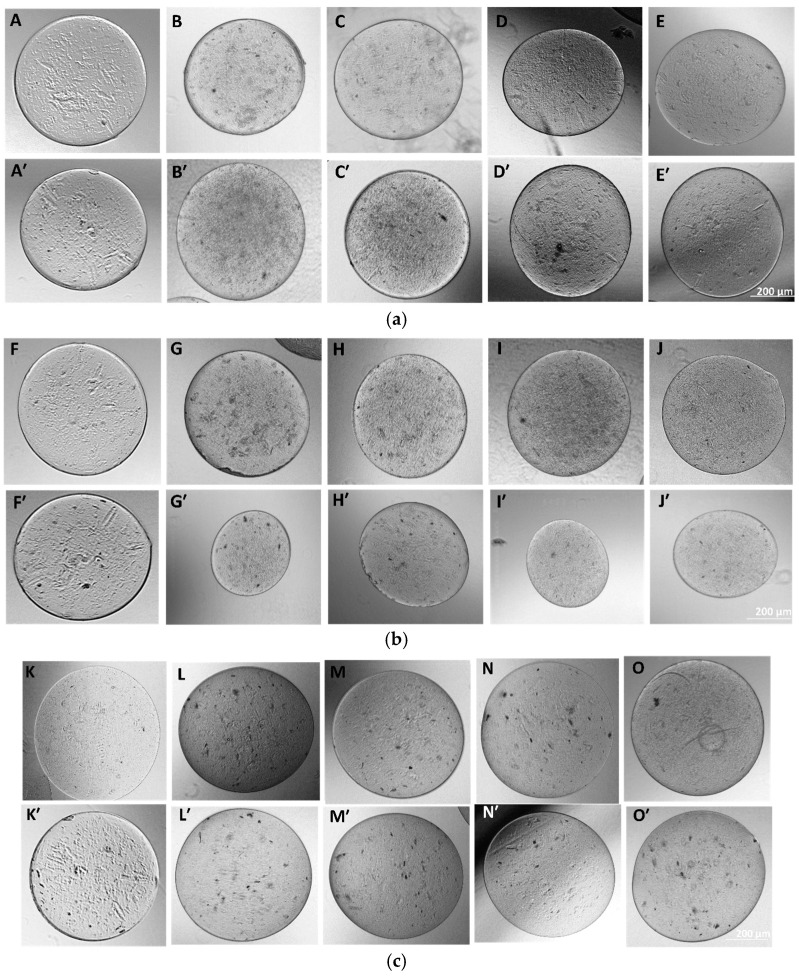
(**a**) Morphology of as-fabricated AAA and APG microcapsules in DI water. Images (**A**–**E**) represent the morphology of the microcapsules AAA and APG 00, 10, 20, and 50, respectively, at day 1, and images (**A’**–**E’**) represent the same at day 3. (**b**) Fabricated APG microcapsules in complete media. (**F**–**J**) represent day 1 microcapsules of AAA, APG 00, 10, 20, and 50, respectively, and (**F’**–**J’**) represent the same on day 3. (**c**) Fabricated APG microcapsules in PBS. (**K**–**O**) represent the day 1 microcapsules of AAA, APG 00, 10, 20, and 50, respectively, and (**K’**–**O’**) represent the same but at day 3.

**Figure 10:**
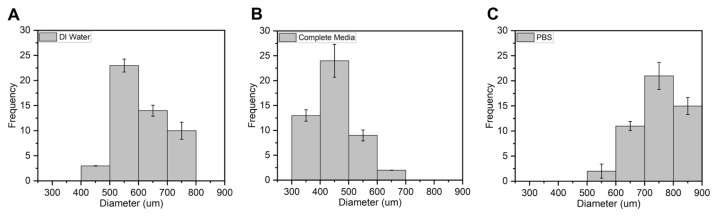
Average size distribution of APG-50 microcapsules in (**A**) DI water, (**B**) complete media, and (**C**) PBS n = 50 on day 3.

Microcapsules were successfully fabricated, immersed in their respective solution, and further analyzed. The optical images of APG representing their different morphologies in each testing solvent environment for days 1 and 3 are shown in Figure 9a–c. The size distribution plot of the microcapsules (n = 50) for day 3 is shown in Figure 10.

The average diameters of the APG-50 microcapsules are expressed quantitatively in a frequency distribution as shown (Figure 10). APG-50 microcapsules were chosen as they performed the best in terms of viability and mechanical strength. From the graph, it was observed that the size of the microcapsules in all solutions ranged between 300 and 800 μm with an average size of 600 μm for the microcapsules in DI water, about 450 μm for those in complete media, and about 750 μm for those in PBS. These changes in sizes could be a result of the entry or escape of the revolving fluid environment of the microcapsules.

**Figure 11:**
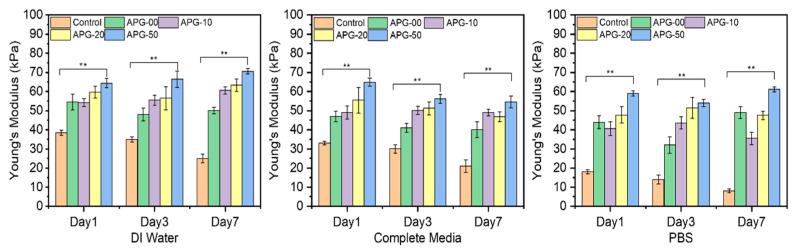
The average Young’s modulus of the microcapsules (n = 5) incubated in the different solvents, which are DI water, complete media, and PBS at days 1, 3, and 7. Statistical analysis was performed using the one-way ANOVA with Tukey’s post hoc method, and the data are expressed as the mean ± SD; n = 3 per group for cell viability test (where ** *p* < 0.01).

Figure 11 further illustrates the mechanical properties of these microcapsules in their respective solvents. The plot demonstrates a significant enhancement in the mechanical performance of the nanofibril-filled microcapsules, regardless of the solution environment, compared to the pure alginate hydrogel microcapsules. Here, the data indicate that the APG-50 microcapsules exhibited a higher Young’s modulus as compared with other groups. This higher Young’s modulus in APG-50 microcapsules is attributed to their stiffer structure, resulting from faster and complete gelation due to purely electrostatic interactions between APG-50 and calcium ions. In contrast, a slower formation of bonds or interactions between calcium ions and pure alginate hydrogel leads to incomplete gelation, requiring slightly less force for the same displacement. The histogram illustrates significant improvements in the mechanical properties of the nanofibril-filled microcapsules regardless of the solution environment compared to the pure alginate hydrogel microcapsules in the different solvent solutions after immersing for different time points (1, 3, and 7 days). This improvement can be attributed to the dual role of the electrospun nanofibers, which function both as fillers in the void spaces and as reinforcing scaffolds within alginate hydrogel [42]. By filling the voids, the nanofibers create a physical network that better distributes and resists the external forces, leading to improved mechanical strength. Additionally, the hydrogen bonding between the amino groups of gelatins and the hydroxyl and carboxyl groups of sodium alginate may further contribute to the enhanced mechanical strength of PCL–gelatin nanofibril-blended alginate hydrogel [43].

## 4. Discussion

The nanofibers of PG were fabricated using electrospinning and characterized for their properties. The fiber diameter averages recorded for the different PG samples showed that the increasing gelatin content reduced the fiber diameter compared to the non-gelatin-containing nanofibers. This reduction could be because the blended PG solution was made up of both synthetic material (PCL) and natural material (gelatin), making the electrospinning solution more electrically conductive; as such, causing the formation of thinner fibers, hence the thinner or reduced diameters. A reduction in fiber diameter has been reported with PCL and other natural polymers such as chitosan in other published works [29,44]. The size or diameter measurements of the nanofibers were essential before cryogrinding because it helps to know the initial sizes of the fibers and how much more these fibers need to be broken down for easy encapsulation with alginate. Also, lower fiber diameters corresponded to a higher porosity. The average porosity percentage of the PG fibers ranged between 58 and 73%, which makes them pliable for cell culture study. Biomaterials for biomedical application should be porous to ensure metabolites and cell nutrients exchange as well as promote cell adhesion and growth tendencies. The scaffold’s pores must also be connected to promote morphological patterning and offer sufficient surface area for homogenous cell distribution.

In the analysis of the FTIR spectra plot, the addition of gelatin with PCL revealed significant characteristic peaks at 1532 and 1638, especially with the PG-20 and PG-50 scaffolds compared with PG-10. These peaks correspond to amine and amide groups of gelatins, confirming the presence of gelatin in all PG samples.

Wettability has been recognized as one of the most important properties of fibrous materials. This is because it helps to determine the hydrophilicity of the testing material for possible good cell expansion. In the determination of the hydrophilicity of the sample, a sample with a contact angle above 90° was considered hydrophobic, whereas a contact angle below 90° was hydrophilic [45]. From the contact angle measurement results, the incorporation of gelatin increased hydrophilicity compared with the pure PCL scaffold. PG-50 had more gelatin; therefore, it was considered as the most hydrophilic, as it recorded the lowest contact angle measurement, as indicated in Table 3. The mechanical properties of materials, in general, for biomedical applications are crucial to boosting decisions on materials’ ability to endure physicochemical changes and mechanical forces in the body. The overall mechanical results’ outcome showed a significant increase in the mechanical properties of the fibers as gelatin content increased. Pure PCL exhibited the highest strain compared to the gelatin-containing fibers. The greatest strength was experienced by the gelatin-containing fibers, as the numbers represent a significant drift from the pure PCL sample. Although it was anticipated that a higher gelatin content would result in improved mechanical properties, the YM data showed that the PG-20 sample was the hardest among all the PG samples. This may be attributed to unbalanced material distribution, thus, an unparalleled testing mechanism.

The biocompatibility study is relevant to determine the materials’ compatibility with cells. The cell viability and proliferation percentages of NIH-3T3 cells in this study were more than 95% on all samples. The enhancement in viability can be attributed to the presence of gelatin, a natural polymer which subsidizes PCL hydrophobicity. The availability of the binding site of gelatin within PCL scaffolds facilitates cellular adhesion, proliferation, and the spreading of NIH-3T3 cells. The growth and proliferation of NIH-3T3 cells are the indications of the deposition of extracellular matrices, which could have an effect on cell differentiation and tissue regeneration [46,47]. Notably, the high proliferation tendencies of NIH-3T3 cells on the PG sample specifies that the bioactive meshes improve the regeneration of NIH-3T3 cells for wound repairing. The observations recorded for the LDH assay also demonstrated that the PG samples showed no cellular toxicity for the different days of cell cultivation. The toxicity plot confirms that a more viable cell–material sample as seen in Figure 4A corresponds with a less significant toxicity as shown in Figure 4B. The measured LDH level values were found to be statistically highly significant between the PG-00 and PG-50 groups (** *p* < 0.01) at days 2 and 3 while comparing to PG-10 and PG-50 (* *p* < 0.5) at day 2. The excellent biocompatibility and cytocompatibility of the scaffolds PG-10, PG-20, and PG-50, compared to PG-00, upregulated the cellular growth, proliferation, and spreading of the NIH-3T3 cells. The structural framework mimics the ECM that could remodel tissue and inspire tissue regeneration for wound healing. The live/dead fluorescence images additionally demonstrated that the different samples have excellent biocompatibility and bioactivity to NIH-3T3 cells. The highest density of green fluorescence in Figure 5 shows the live and healthy cells, while the few red fluorescences represent the dead cells in each scaffold sample, indicating that the scaffolds are compatible with cells. More than 95% of cells were observed to survive incubation with different scaffolds, suggesting outstanding cytocompatibility to the NIH-3T3 cells that accelerate cell growth, proliferation, and survivability. Cellular activities are generally influenced by the fibrous mesh that has an excellent capability to mimic the natural microenvironment for cells and easily promote tissue healing.

The microencapsulation of materials is essential to preserve and strategically deploy certain nutrients to target areas. Cellular microencapsulation comprises the immobilization of cells in a biomaterial with the potential to overcome recurring limitations, such as immunogenicity, through physically blocking molecules larger than a perilous size and allowing the diffusion of small-sized molecules [48]. Cell encapsulation efficiencies have been proven to be effective, as has been published in previous work [39]. The design and development of tissue engineering techniques and injectable medication delivery systems depend heavily on the choice of biomaterial. The most commonly used materials are hydrogels, which sometimes shelter cells from needle injection [49] and give them support. Parameter consideration in terms of applied voltage, choice of biomaterial, the working distance, or the distance over which the potential difference is applied, needle gauge, and flow rate are key to successfully fabricating desired microcapsules for its use. Increasing voltage has an apparent effect in increasing the size of the microcapsules, and as such, voltage was maintained at 5 kV for this work after a few experimental trials. Microcapsules with a limited particle size distribution can arise from partial or intermittent jet formation at low applied voltages but sustained and continuous jet formation at higher applied voltages. The diameter of the needle also affects the diameter of the microcapsules; for the same operating voltage, a large inner diameter (lower gauge) needle produces microcapsules with a larger diameter than one with a small inner diameter (higher gauge). There is a working distance threshold, both above and below, for which microcapsule formation is impossible. Fundamentally, using longer working distances dramatically lowers cell survival. The effect of the working distance was highlighted in the works of Paletta et al., where after the distance between the needle and the collecting dish was raised from 6 to 11 cm, a 10% decrease in cell viability was recorded, which means a longer distance causes cell-containing droplets to evaporate more quickly, which raises the concentration of salt and reduces cell survival. It is now possible to modify materials to regulate the encapsulated cells’ growth and differentiation or the kinetics of drug release thanks to the development and optimization of bioactive biomaterials for cell encapsulation [50]. For the easy and better encapsulation of the fibrils with alginate for this work, the nanofibers were broken down into small, fine particles utilizing the cryogrinding technique. The images shown in Figure 6 demonstrate that the cryoground was not uniform, as there were both longer and shorter pieces of fibers. These images only confirm the effectiveness of cryoground in reducing the size of nanofibers for further studies. To reduce the fiber sizes further, probe sonication was adopted, which was effective. SEM analysis and the optical images of the cryoground PG particles confirm that the cryogrinding process was successful in converting electrospun nanofiber mesh into short fibrous particles of about 5–30 μm from the initial 80 to about 140 μm. After the successful cryogrinding process, the microcapsules were fabricated. Electrospraying has been used extensively for the mass fabrication of uniform micro-particulates such as microcapsules over the past two decades [51]; as such, this necessitated its use for our study. The optical representation of these microcapsules gives an indication that these microcapsules can survive in different solvent environments. A closer look inside the microcapsules showed effective distribution of the nanofibrils inside the hydrogel microcapsules, giving an indication of the potential of actively encapsulating other important materials that can promote cell viability and improve the mechanical strength. This can further be optimized if tunable parameters are well investigated. The optical images show that microcapsules in DI water remained relatively the same shape and size even after day 3. The same cannot be said for the microcapsules in complete media, which saw a drastic reduction in the size of the microcapsules in the complete media up to day 3. This could be associated with the possible exchange of water or minerals with the environment, such as water or media draining out of the microcapsules, thus reducing the size. It is thus clear that osmotic potentials in different media result in changes in the microcapsule sizes over different time periods. However, the strength of the microcapsules in the complete media was not significantly affected. There was an absolutely opposite outcome with the microcapsules displaced in PBS. These sizes appreciated, and this could also be explained by a possible in-take of fluid from the solvent environment into the microcapsules. These different microcapsules were further studied for their mechanical properties.

Figure 11 further shows the mechanical standpoint of these microcapsules in their respective solvents. The plot shows an effective and significant improvement in the mechanical properties of the nanofibril-filled microcapsules regardless of the solution environment compared to the pure alginate hydrogel microcapsules. This improvement in the mechanical property can resolve the limitations of alginate hydrogel and further developments in the area. This encapsulation mechanism, therefore, can be considered for future 3D works.

The overall outcome of the PG data so far showed that these PG scaffolds may be utilized to develop a porous tissue engineering construct that resembles the fibrous extracellular matrix (ECM) present in tissues [52]. Polymer microcapsules generally demonstrate high efficiency compared to other microencapsulation methods such as air-suspension microencapsulation, spray drying, and freeze drying [53]. Polymer microcapsules show excellent encapsulation efficiency, often ranging from 73.9% to 95.9% [54]. For example, ethyl cellulose and cellulose acetate butyrate polymer microcapsules achieved encapsulation efficiencies greater than 87.21% [55]. The choice of specific performing polymers for microencapsulation depends on the study’s purpose, whether it is for mechanical, physicochemical, or biological reasons. Even though many natural polymers such as chitosan and alginate tend to gel under circumstances that are conducive to mammalian cell encapsulation, issues like batch-to-batch variability and residual components from the original source trigger an immunological reaction when implanted [56].

Synthetic polymers like PCL, polyethylene glycol (PEG), and polyacrylates offer greater mechanical and chemical stability over traditional materials. There is an increased reproducibility due to minimized batch-to-batch variation [57,58]. In other runs, PEG, whose molecular weights were steadily increased in a test, had tensile strength ranging from 0.60 MPa to 2.20 MPa. PCL had a relatively better tensile strength, which for our work recorded 9 MPa for our fibrous scaffold. This figure indicates the stretching and expansion capacity of PCL for our work over PEG [59]. Polyacrylate can achieve high encapsulation efficiency for an optimized system, as recorded from the works of Rossi. However, acrylate has a faster degradation and will be the choice for relatively faster degradation applications [60].

PCL demonstrates good encapsulation efficiency, with one study reporting 75% for naringin encapsulation [61]. Its survival in the different solvents for our work makes it ideal for consideration in further works. Combining synthetic with natural polymer ensures that shared properties stabilize potential limitations associated with individual polymers. So far, the APG microcapsules did not have changes in structure even after day 7 in the different solutions, except in the complete media. The change in the complete media could be an exchange of nutrients between the media and the microcapsules, as such, reducing the size of the microcapsules after day 3.

Table 6 shows a comparative analysis of our work with the published literature. As can be seen, the mechanical strength is substantially higher compared with other polymers. Similarly, our formulation resulted in >100% cell viability, which was superior to other polymers. Thus, fibril-laden PCL microcapsules showed superior properties and cellular viability toward a broad range of biomedical applications such as drug delivery carriers and organoids.

Electrospraying and electrospinning techniques address scalability concerns and can now be utilized for the large-scale production of nano- and microscale materials [68]. The adaptation of electrospinning and cryogrinding techniques for large-scale production is not without compromising the fiber morphology, mechanical properties, or cytocompatibility. However, implementing high automated-throughput electrospinning systems with multiple nozzles and controlled fiber deposition can significantly increase production capacity. High-throughput systems, such as industrial-scale electrospinning machines, can now use up to 5500+ needle emitters and process up to 60 L of solution, enabling 24/7 production [69]. Scaled-up multiple-nozzle electrospinning technologies have been successfully used in the pharmaceutical industry after adjusting process parameters and modifying spinnerets [70]. Additionally, adopting continuous cryogenic grinding systems with precise temperature control can maintain uniform particle size and prevent degradation during bulk processing. Refining processing parameters (e.g., polymer concentration, flow rate, and electric field strength) through the design of experiments (DOEs) can ensure efficiency at scale [71]. Evaluating batch-to-batch variability in fiber diameter, porosity, and surface area [61] is essential in translating this fabrication process to industrial or clinical settings. This is possible by developing standardized protocols for fiber synthesis, including fixed polymer ratios, temperature settings, and environmental conditioning. Also, integrating sensors and image analysis systems for the real-time monitoring of fiber morphology and diameter during production will be considered. Implementing batch-wise quality control tests, such as FT-IR analysis and mechanical property assessments, to verify consistency will be considered as well. Exploring methods for ensuring sterility and maintaining biocompatibility during upscaling, especially for clinical use will be essential. Implementing a cleanroom environment and aseptic handling during fiber fabrication, exploring methods such as gamma irradiation or ethylene oxide treatment for bulk sterilization while preserving material integrity, and pre-screening materials through sourcing medical-grade PCL and gelatin to ensure baseline biocompatibility and safety can be beneficial.

Sample size was considered in each of our studies ranging between 3 and 100 depending on the test. For instance, in our contact angle measurement, a sample size of three (n = 3) was considered. This means the average and standard deviation were calculated based on three independent measurements. While this provides a basic estimate of central tendency and spread, a larger sample size would be more robust for statistical significance and reliability. Our data show a decreasing trend in contact angle values from PG-00 to PG-50, indicating increased hydrophilicity with increasing PG content. A lower contact angle suggests improved wettability, which could be beneficial for cell attachment and nutrient absorption in scaffold applications. The decreasing contact angle with increasing PG content suggests a consistent modification effect on the scaffold surface properties. The standard deviations indicate the variability of measurements within the triplicate samples. For instance, PG-00 has a higher standard deviation (6.14) compared to PG-50 (3.88), suggesting greater variability in the measurements for the PG-00. These differences can be explained due to a variety of reasons. It could be a lack of overall even distribution of the nanofibrils in the microcapsules or the lack of proper mixing of the samples of PCL and gelatin prior to mixing with alginate hydrogel. The standard deviations highlight the importance of repeatability in future work and the possible considerations of minor inconsistencies in scaffold fabrication or measurement conditions. In summary, variability was accounted for by repeating the experiment; however, for future works, more sample sizes need to be considered for less variations.

## 5. Conclusions

This research aimed to synthesize a 3D platform construct of alginate hydrogel filled with cryoground nanofibrils of PCL and gelatin, which can improve alginate hydrogel limitations for biomedical applications. The electrospinning technique was first adopted to fabricate the electrospun nanofibers. The electrospun nanofibers were characterized for fiber morphology, FT-IR, mechanical properties, and cytocompatibility. The pure PCL (PG-00) nanofiber’s surface area per unit weight (m^2^/g) was estimated as 1.8 ± 0.51 as compared to the nanofiber containing an equal proportion of PCL–gelatin (PG-50), which was estimated as 0.9 ± 0.54. The porosity of the PG fibers ranged from 58 to 72% from the pure PCL sample (PG-00) down to the PG-50 sample. Thus, it was concluded that the higher the surface area per unit weight, the lower the porosity, and vice versa. Overall fibrous results showed an improvement in the properties of the electrospun PCL fibers with the addition of gelatin. Even though the specific purpose determines what ratio is best, overall, the PG-50 combination was the best, since it showed the most viability/less toxicity at the fibrous testing and showed more mechanical capability as microcapsules. Alginate microcapsules have shown enormous potential in various applications, especially in tissue engineering and regenerative medicine. The integration of electrospun fibrils with alginate microcapsules represents a promising strategy for enhancing mechanical and biological limitations experienced by alginate hydrogels. The synergistic combination of fibrin’s bioactivity with alginate’s biocompatibility offers numerous advantages for biomedical applications such as drug delivery and tissue engineering. While challenges remain, ongoing research efforts hold great promise for advancing this technology toward clinical translation, improving patient outcomes. The development of the 3D structure closely mimics the microenvironment and will advance limitations thereof. Additionally, the development of advanced fabrication techniques, such as 3D bioprinting or microfluidic-assisted assembly, may enable precise control over scaffold architecture and cellular organization, further enhancing tissue functionality.

## Figures and Tables

**Table 1:** PG sample designation and composition.

Sample Name	Proportion of PCL: Gel (*w*/*w*) %
PG-00	100:0
PG-10	90:10
PG-20	80:20
PG-50	50:50

**Table 2:** APG microcapsule designation and composition.

Sample Name	Volume of 2% Alginate (mL)	Amount of Fibers Used (mg)	Proportion of PCL–Gelatin (*w*/*w*)
**AAA** (Alg-100%)	700	-	-
**APG-00** Ratio (Alg-70%: PCL-30%)	700	4.2	100:0
**APG-10 (Alg-70%)** Ratio (PCL-90%: Gel-10%)	700	4.2	90:10
**APG-20 (Alg-70%)** Ratio (PCL-80%: Gel-20%)	700	4.2	80:20
**APG-50 (Alg-70%)** Ratio (PCL-50%: Gel-50%)	700	4.2	50:50

**Table 3:** Rectangular PG scaffold’s dimensions (40 mm × 15 mm) with equivalent porosity evaluation. The table shows the numeric comparison of the thicknesses, surface areas, and surface area per unit weight of the different meshes (PG-00 to PG-50).

Mesh	Thickness (mm)	Surface Area (m^2^)	Surface Area per Unit Weight (m^2^/g)	Porosity (%)
PG-00	0.155 ± 0.0063	0.144 ± 0.05	1.8 ± 0.51	60.3 ± 5.3
PG-10	0.149 ± 0.0048	0.113 ± 0.03	1.3 ± 0.37	58.8 ± 2.9
PG-20	0.163 ± 0.0041	0.107 ± 0.04	1.3 ± 0.62	64.2 ± 6.2
PG-50	0.194 ± 0.0093	0.091 ± 0.05	0.9 ± 0.54	72.3 ± 4.9

**Table 4:** Surface wettability analysis of PG samples by contact angle measurement. The contact angle measurement between the mesh type and the drop of water for samples PG-00 to PG-50 showed decreases in the contact angles as gelatin content increases, which means increases in the hydrophilicity property.

Scaffold Name	Average Contact Angle (Angle + SD)
PG-00	122 ± 6.14
PG-10	78 ± 3.42
PG-20	72 ± 5.11
PG-50	61 ± 3.88

**Table 5:** Mechanical analysis summary table of PG samples. This table shows the numeric representations of the ultimate tensile strength, Young’s modulus, and the breaking strain of the different PG samples’ runs.

Sample	Ultimate Tensile Strength (MPa)	Young’s Modulus (MPa)	Breaking Strain (mm/mm)
PG-00	9.31 ± 0.61	18.33 ± 1.53	10.3 ± 1.22
PG-10	22.26 ± 2.25	33.47 ± 3.21	5.32 ± 0.37
PG-20	22.84 ± 2.21	32.89 ± 2.78	4.04 ± 0.51
PG-50	25.59 ± 1.94	32.66 ± 3.01	6.15 ± 0.74

**Table 6:** Comparative analysis of other published works of PCL and other natural polymers.

Hydrogel	Young’s Modulus (MPa)	Cell Viability	References
PCL–gelatin fibril/alginate	~32.66	>100%	Our work
Chitin/PLGA/alginate	~0.0423	-	[62]
Chitosan/PCL/TFNA	~4.37	>90%	[63]
Alginate/sodium cellulose Sulfate	-	>70%	[64]
STMS-GA@PDA	≤7.3 MPa	-	[65]
GelMA/GOX/BSA	~0.0025	>97%	[66]
PLGA and alginate	~2.1	>80%	[67]

## Data Availability

The original contributions presented in this study are included in the article. Further inquiries can be directed to the corresponding author.
